# Advanced low-power filter architecture for biomedical signals with adaptive tuning

**DOI:** 10.1371/journal.pone.0311768

**Published:** 2025-01-23

**Authors:** Ramasamy Srinivasagan

**Affiliations:** Computer Engineering, CCSIT, King Faisal University, Al Hufuf, Kingdom of Saudi Arabia; Universiti Teknologi Malaysia, MALAYSIA

## Abstract

This paper presents a low-power, second-order composite source-follower-based filter architecture optimized for biomedical signal processing, particularly ECG and EEG applications. Source-follower-based filters are recommended in the literature for high-frequency applications due to their lower power consumption when compared to filters with alternative topologies. However, they are not suitable for biomedical applications requiring low cutoff frequencies as they are designed to operate in the saturation region. The major contribution in this work are the filter is made to operate in the weak inversion zone to reduce the area needed for the capacitor and the amount of power dissipated. Process variation is one of the major issues in the weak inversion regime. To overcome this, a unique method of compensating against fluctuations in process, voltage, and temperature is put forth based on magnitude comparison is another contribution. Key findings from post-layout simulations and experimental measurements demonstrate that the filter achieves a tunable cutoff frequency range of 0.5 Hz to 150 Hz, with a total power dissipation of only 6nW at 150 Hz. The design occupies a compact silicon area of 0.065 mm^2^ and offers a dynamic range of 75 dB. The measured results indicate that for a 300 mVpp signal swing, the top bound on THD is -40 dB. The filter’s robustness against process, voltage, and temperature variations is validated through on-chip tuning using a current steering DAC, ensuring stable performance across different operating conditions. These results make the proposed filter a promising candidate for low-power biomedical devices. The recommended filter is developed and implemented using UMC-0.18μm CMOS technology with a 1.0V supply, and the IC is tapped out using an MPW run of Euro practice IC services.

## I Introduction

The development of battery-operated portable devices has revolutionized various fields, including biomedical instrumentation. Specifically, devices such as personal heart rate monitors based on electrocardiograms (ECGs) and electroencephalogram (EEG) systems are gaining popularity due to their non-invasive nature and effectiveness in monitoring physiological signals. However, designing low-power, highly tunable filters for these applications presents significant challenges. One of the primary challenges in designing filters for ECG and EEG applications is achieving both **low power consumption** and **high tunability**. These applications require filters that can process low-frequency signals within a narrow bandwidth, typically from 0.5 Hz to 150 Hz [1], while maintaining a dynamic range capable of handling weak signals with amplitudes ranging from a few microvolts to millivolts. Low power consumption is critical for prolonging battery life in portable devices, whereas high tunability is necessary to adapt to various signal conditions and mitigate process variations.Traditional filter architectures, such as Gm-C and OTA-C filters, have been used for biomedical applications, but they often struggle to balance power efficiency and performance. Many of these designs operate in the strong inversion region, which consumes more power and requires large capacitors to achieve the desired frequency response. In contrast, operating transistors in the **weak inversion region** allows for significant power savings, but it introduces challenges in maintaining linearity, reducing noise, and ensuring robustness against process, voltage, and temperature (PVT) variations.

**Fig 1:**
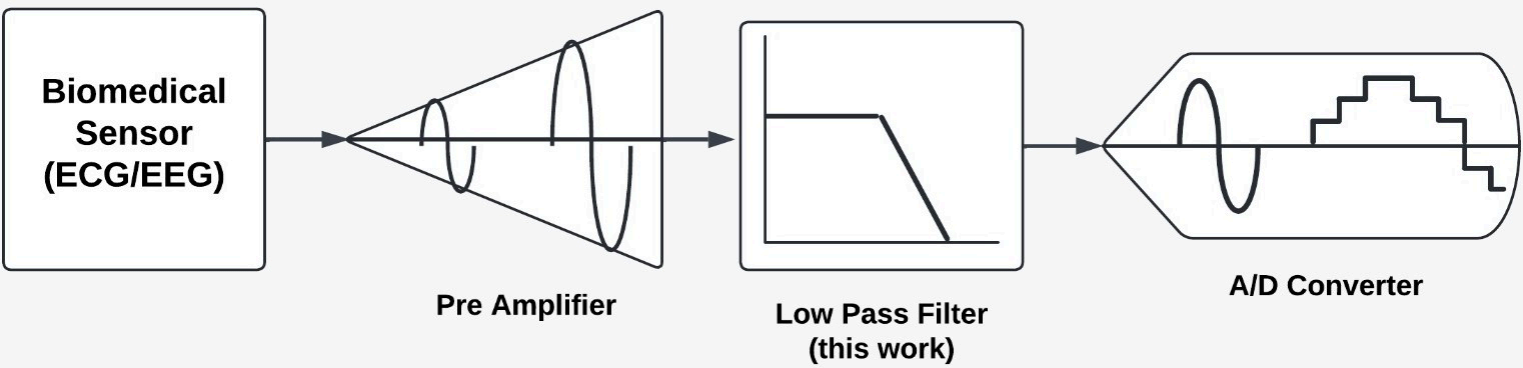
Biomedical signal processing system.

To address these challenges, recent research has focused on developing new architectures and design techniques that strike a balance between power efficiency and performance. The electroencephalogram (EEG) is a tool used in various clinical and scientific applications to capture the electrical activity of the human brain. The basic EEG signals consist of four bands of brain waves: δ (1–4 Hz), θ (4–8 Hz), α (8–13 Hz), and β (13–40 Hz), which constitute the basic EEG signals [2]. These signals, which are very faint (2 to 200 μV) and show continuous oscillating curves, must be acquired in noisy surroundings.Small physical size and low power consumption are crucial for portable equipment like ECG and EEG systems. Additionally, to process the weak signal further, it must first be amplified and filtered (amplitude less than a few millivolts). For an accurate diagnosis, filters operating in the frequency range of a few hertz to a few hundred hertz are particularly important. For the analog front-end circuits and digital processors to be incorporated into a low-voltage system-on-a-chip (SoC) system, they need to function at a low supply voltage [3]. Fig 1 shows the biomedical signal processing system’s block diagram. It consists of a low-pass filter, a preamplifier, an analog-to-digital converter, and biomedical sensors for ECG and EEG signals.

Our proposal for the aforementioned application is a continuous-time, programmable, low-power low-pass filter. Large constants necessitate big capacitors and silicon areas for low-pass filters used in biomedical applications. A low-pass filter based on switched capacitors (SCs) has been suggested in the literature [4–6] for the use of an ECG monitoring system. However, sample-hold circuits in SC filters are inappropriate for applications that need time constants of a few milliseconds or less due to the rise in leakage currents in the advanced CMOS process [3]. In [7], a Gm-C filter based on a FGMOS transistor operating in the weak inversion region is suggested for use in biological applications. Unfortunately, because of the mismatch in capacitor ratios, it experiences a rise in the second-order harmonic. Sub-nS OTAs in [8] intended for biological applications are implemented in 0.8μm technology with series-parallel current mirrors. Nevertheless, in sophisticated operations, the OTA offsets with this method increase. There have been reports of Gm-C filers functioning in mild or moderate inversion areas [9–11]. However, distortion is incorporated into the voltage-to-current conversion process in these Gm-C filters. This issue may be resolved by utilizing a source-follower-based continuous time filter, and in [12], a low-pass filter with a high cut-off frequency is suggested for use in a WLAN application. In a strong inversion zone, the aforementioned filter functions. Because of its huge transconductance, it cannot be directly employed for biological purposes without the need for very large capacitors. C. Sawigun et al. propose in [13] a second-order Gm-C filter working in the subthreshold zone using negative feedback with only seven transistors running at low voltage. On the other hand, the filter needs extremely low bias currents between nA and pA which is exceedingly challenging to produce on the chip.

Furthermore, only a maximum of 30% frequency change is allowed by the spread of circuit characteristics, which restricts tunability. Widely variable 4th order Butterworth Gm-C filter by Krishna et al. [14] that operates in the weak inversion area without the need for common mode feedback due to its differential structure, which was constructed using 0.18 μm CMOS technology. They demonstrated a similar dynamic range and Fig of merit in comparison to the most advanced low-frequency filters available. Post-layout simulations are used to illustrate the outcomes. Zhang et al. [15] presented a further, largely adjustable OTA-based current steering integrator-based filter for biomedical signal filtering. The chip size was around 450 μm x 150 μm, and it was manufactured using a conventional 0.18 μm CMOS process. The architecture may be utilized for both low-pass and notch filters. A 4th-order cascade flipped source follower filter (CFSF) by Dhiksa Thakur et.al [16] was proposed for wearable biological healthcare applications. The architecture was designed in a standard 0.18 μm CMOS process. The post-layout simulation using BSIM3v3 MOS models reported good metrics in terms of area and power, however the dynamic range is poor. Saleha Bano et.al [17] proposed, flipped voltage follower based fourth order filter and its application to portable ECG acquisition system. The Fig of merit reported was good, but again this is not a measured results. Montree Kumngern et.al [18], proposed fully differential fifth order dual notch low pass filter for portable EEG system, which is not power efficient and area is not reported.

Filters for biomedical applications require very large capacitors due to large the transconductance. To reduce the capacitors required for the filter to be of the order of a few tens of pico Farads, the source follower-based filter operating in a weak inversion saturation region is proposed in this paper. A crucial factor to take into account when implementing ting the Gm-C filter is modifying the filter parameters, specifically C and Gm. Because they are influenced by temperature fluctuations and process parameter limitations, they are not perfectly controllable in CMOS technology. Furthermore, the matching qualities of MOS devices operating in the weak inversion area are not good. An onchip-tweaking approach is warranted for each of these issues. This work proposes a tuning mechanism based on magnitude comparison for this purpose.

The major contributions of this work are:

1. Tapped-out source follower-based filter architecture operating in a weak inversion regime for biomedical signal filtering in the 180 nm CMOS process.
2. Tested on a chip tuning scheme and validated with measurements to mitigate the PVT process voltage and temperature variations, which were proposed in [19].

The remainder of the paper is structured as follows: The second-order low-pass filter based on the source follower that operates in the weak inversion zone is described in Section II. Section III discusses the center frequency’s tuning technique and programmability. Section IV presents the post-layout simulation results, which are supported by experimental data and demonstrate the tuning capability’s robustness. Section V offers the conclusions.

## II The proposed second-order low-pass filter for biomedical applications

To begin with, Fig 1 illustrates a biomedical signal processing system designed to capture and process signals from biomedical sensors, such as EEG (electroencephalogram) and ECG (electrocardiogram).

This system comprises several key components, each playing a crucial role in ensuring accurate signal acquisition and processing:

*Biomedical Sensor (EEG/ECG)*: These sensors are responsible for detecting physiological signals from the body. EEG sensors monitor brain activity, while ECG sensors record heart activity.

*Preamplifier*: The preamplifier amplifies the weak signals received from the biomedical sensors. This amplification is essential to enhance the signal strength while maintaining the integrity of the original signal, preparing it for further processing.

*Low Pass Filter (This Work)*: The low pass filter, designed in this work, is a critical component that allows signals with frequencies below a certain threshold to pass. This filtering is crucial for isolating the relevant biomedical signals from high-frequency noise and artifacts, thus improving signal clarity and quality.

*A/D Converter*: The analog-to-digital converter (A/D converter) transforms the filtered analog signals into digital form. This conversion is necessary for the subsequent digital signal processing and analysis, enabling the utilization of advanced computational techniques for accurate interpretation and diagnosis. This system is integral to biomedical applications, providing a foundation for precise monitoring and analysis of physiological signals, which are essential for diagnostic and therapeutic purposes.

### a. Overview of the source follower-based filter

The first proposal for a second-order low pass filter having source follower architecture is in [12]. The following are some advantages of this architecture:

1. It produces a low output impedance and permits the driving of a resistive load with little impact on the linearity and precision of the filter transfer function.
2. The structure’s intrinsic feedback provides improved linearity.
3. Distortion of current mode filters resulting from the voltage to current conversion process is absent.

**Fig 2:**
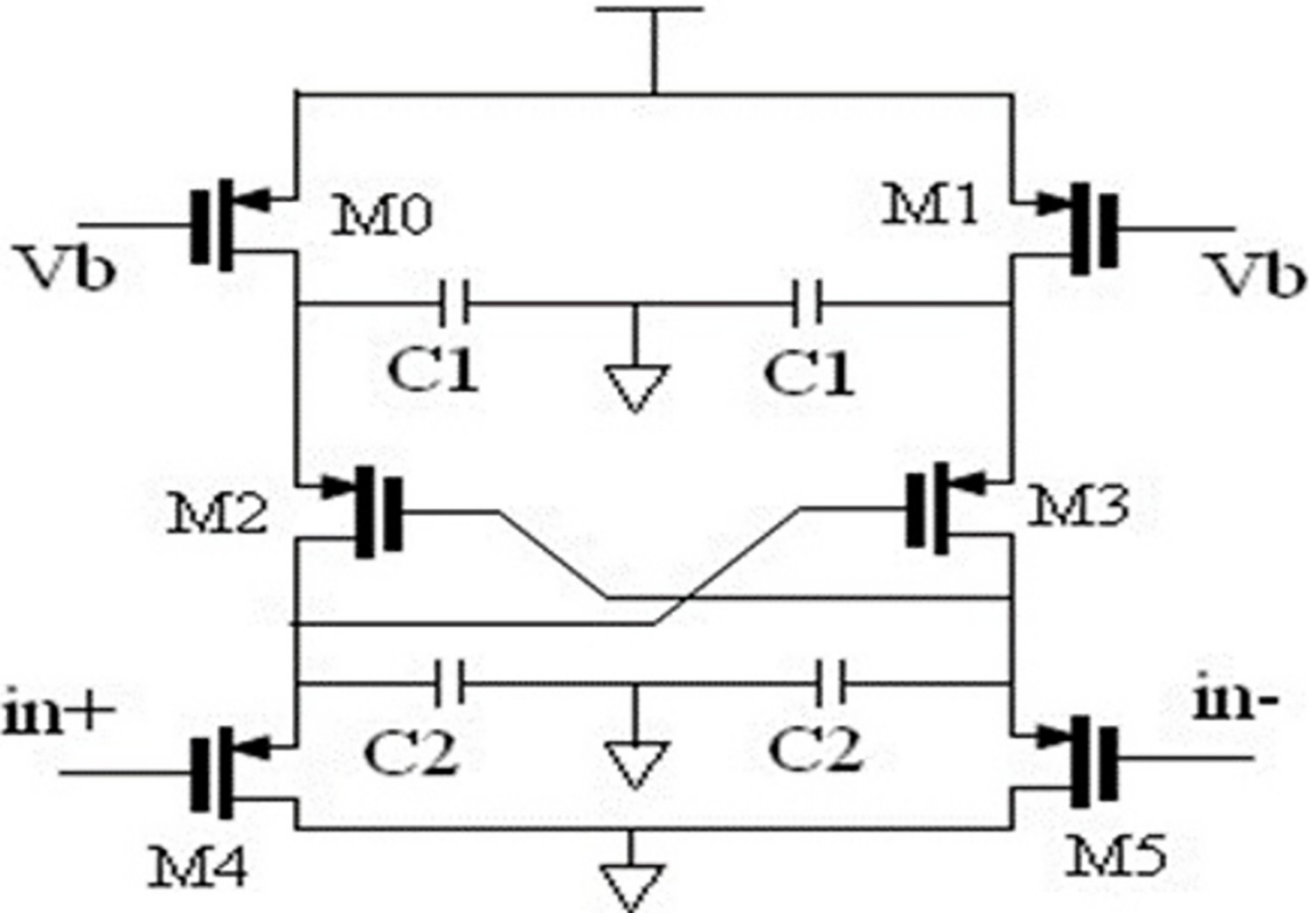
Second-order source follower filter.

Fig 2 illustrates the redesigned filter suggested in [12] that allows tunability and removes bottom plate capacitance. Similar to a "composite" source-follower, it perates with a completely differential structure. It offers an ideal DC gain of unity.

The M0 through M5 transistors are all made to have the same dimensions and hence they draw equal current. As a result, they all demonstrate the same transconductance values. If we assume that the transistor’s output conductance (g_ds_) is significantly less than the transconductance (g_m_), we can show as in [12] that the filter transfer function is that given by Eq (1).

$$H(S)=\frac{1}{s^{2}.\frac{C_{1}C_{2}}{{g_{m}}^{2}}+S.\frac{C_{1}}{g_{m}}+1}$$ (1)

where gm is the transistor’s transconductance and C1 and C2 are the capacitors. Eqs (2) and (3) provide the filter parameters (pole frequency, quality factor, and DC-gain).


$$Q=\sqrt{\frac{C_{2}}{C_{1}}}$$ (2)

$$\omega_{o}=2\pi f_{o}=\frac{g_{m}}{\sqrt{C_{1}C_{2}}}$$ (3)

It is evident from Eq (3), that we can change the operation frequency by adjusting the gm and the capacitor values.The low pass filter in [12] is targeted for WLAN applications. Since the cut-off frequency of this filter is of the order of a few MHz, the gm required for the filter is large and hence the transistors are supposed to operate in a strong inversion saturation region. However, the proposed filter’s transconductance has to be low to obtain a low cut-off frequency for a biomedical filter and hence we operate all transistors in a weak inversion region.

### b. Proposed filter for biomedical application

The modification of the low pass filter shown in Fig 2, for processing biomedical signals such as ECG and EEG is considered next. The cut-off frequencies of this filter vary from 0.5Hz to 150Hz. Assume that the filter is a Butterworth filter with a quality factor of 0.707 and capacitors C1 and C2 have respective values of 20pF and 10pF. The gm required for this frequency range varies from 25pS–to 12nS. To achieve this lower gm value, the transistor is needed to operate in the weak inversion/subthreshold region. The filter’s low-power operation in weak inversion mode is ideal for low-power and low-frequency applications for the following reasons:

1. Devices operating in the weak inversion region have smaller maximum voltage variations between their terminals than those working in the strong inversion zone. As a result, lowering the supply voltage can reduce power consumption
2. Larger capacitance (C_GS_ and C_GD_) and smaller transconductance(g_m_) are needed for the low-pass filters. It is possible to raise the capacitance values by multiplying the Width(W) and length(L) of the apparatus. However, the breadth must be narrow to produce lower gm. The result is an increase in the device’s length. When longer devices are used, there is an improved device-switching effect.
3. The transistor VGS self-biases the output common mode voltage and thus reduces the need for a CMFB (common mode feedback) circuit, thereby resulting in lower power dissipation. Eq (4) describes the (g_m_) of a transistor operating in the weak inversion region.

$$g_{m}=\frac{I_{D}}{n\Phi_{T}}$$ (4)

where “Φ_T_” is thermal voltage and “n” is sub threshold slope factor. I_D_ is the current of a MOS transistor [20] and is given by Eq (5),

$$I_{D}=2nk{\Phi_{T}}^{2}exp\left(\frac{V_{GB}-V_{T0}}{n\Phi_{T}}\right)\left[exp(\frac{-V_{SB}}{\Phi_{T}})-exp(\frac{-V_{DB}}{\Phi_{T}})\right]$$ (5)

where k = β.(W/L) called as transconductance parameter, β = μ. Cox is the process gain factor; “μ” is the mobility of carriers; “Cox” is the oxide capacitance of the transistor. The gate, source, and drain voltages w.r.t. bulk respectively are V_GB_, **V**_**SB**_, and **V**_DB_; **V**_T0_ is the threshold voltage.In the subthreshold zone, **V**_DB_ > 5 Φ_T_ and V_SB_ is 0. Here, Eq (5) reduces to Eq (6).


$$I_{D}=2nk{\Phi_{T}}^{2}exp\left(\frac{V_{GB}-V_{T0}}{n\Phi_{T}}\right)$$ (6)

It is clear from (4) that the current must be adjusted between 30pA to 40nA to get the necessary gm range. V_GS_ is found to be between 100mV to 400mV using Eq (6) and the assumptions that V_T0_ of 0.5V and the transistors W/L to be 1*μ*m /10*μ*m, This necessitates varying the DAC voltage between 600mV– 900mV. The Quality factor (Q) and linearity are taken into consideration when selecting the transistor length for the aforementioned filter. It is demonstrated [12] that the Q factor’s sensitivity to the output MOS capacitance, gds, is that which is indicated in (7)

$$S_{g_{ds}}^{Q}=\frac{\partial Q}{\partial g_{ds}}.\frac{g_{ds}}{Q}\approx -\frac{3}{2}\frac{g_{ds}}{g_{m}}$$ (7)

A small value for g_ds_ is required to make the Q factor independent of any variations in output conductance (g_ds_), Let’s examine how the length of the transistor affects the filter’s linearity. Second-order harmonic HD2, which is entirely differential in the filter construction, is canceled out. When transistors operate in weak inversion, it can be demonstrated that the third harmonic distortion (HD3), assuming it to be the primary contributor to overall harmonic distortion, is provided in Eq (8) at low frequencies.


$$HD3=\frac{1}{6}.\frac{V_{in}^{2}}{{\left(n.\frac{KT}{q}\right)}^{2}}.\frac{1}{{\left(1+\frac{g_{m}}{g_{d0}}\right)}^{2}}\approx \frac{1}{6}.\frac{V_{in}^{2}}{\frac{1}{\lambda^{2}}}$$ (8)

It can be observed from Eq (8), that increasing **“λ”** (channel length modulation coefficient) will increase the linearity. This consequently necessitates lightening the transistors. For the implementation in 0.18μm CMOS technology, the length of the transistor is chosen to be 10μm to achieve a low g_ds_ value and high linearity.

### c. Noise performance of the proposed filter

**Fig 3:**
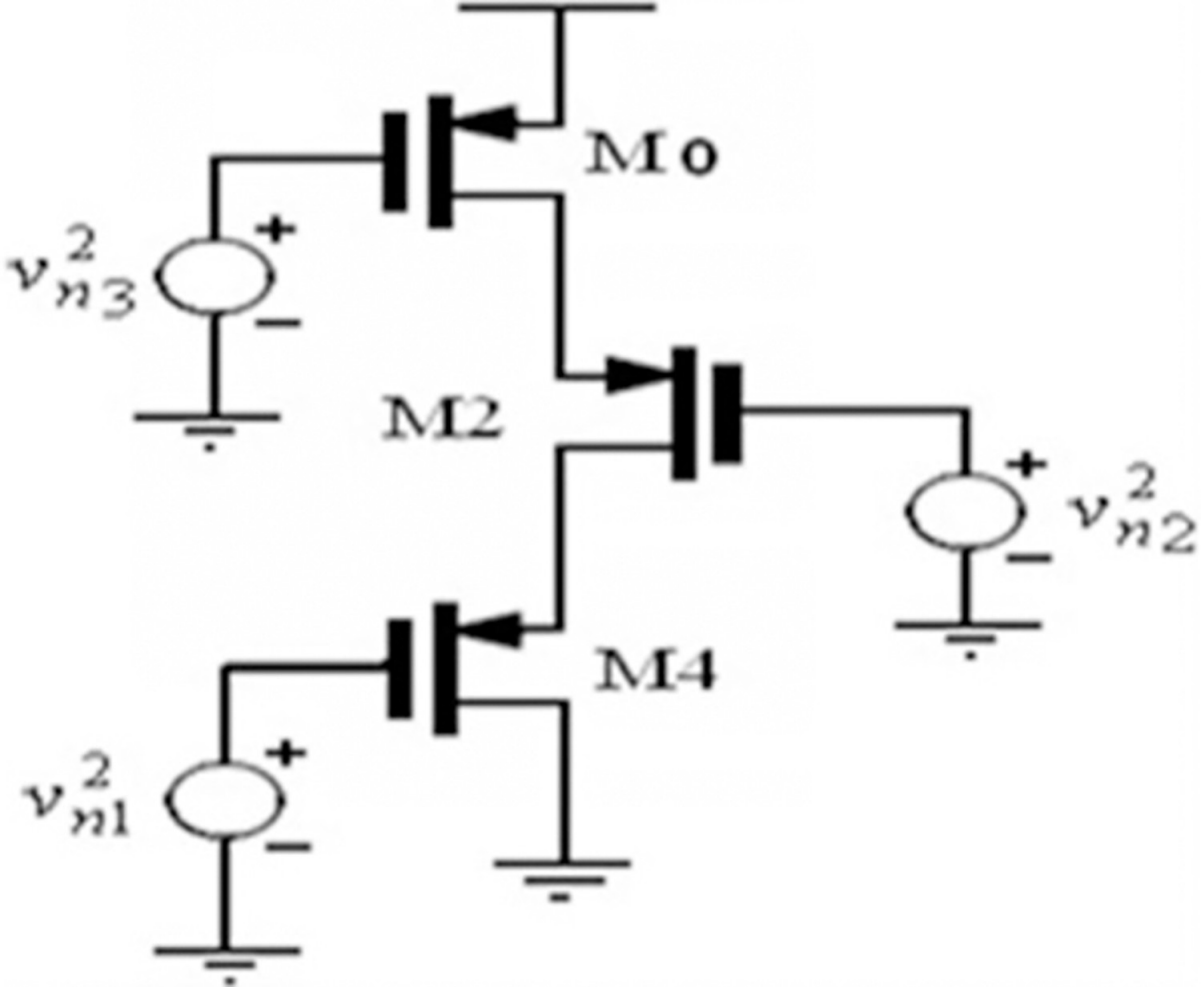
Half-circuit noise model of source follower filter.

The transistor flicker noise component alone dominates at very low frequencies, which correspond to biological signals. PMOS transistors are preferred due to their ability to lower flicker noise and it is inversely proportional to gate area. To reduce flicker noise, the gate area has to be increased. To maintain a low “gm” of the order of nS, the W/L ratio should be low, so transistors with longer lengths and smaller widths are chosen. Using the half circuit noise model shown in Fig 3, second order source follower circuit’s input referred voltage noise can be calculated

**V**_**n1**_^**2**^, **V**_**n2**_^**2**^, and **V**_**n3**_^**2**^ represent mean square noise voltages for transistors M4, M2, and M0, respectively. It should be noted that the dimensions of all these transistors are identical. Eq (9) calculates the mean square input-referred voltage noise (V_n_^2^) caused by a transistor’s flicker noise [17].

$${V_{n}}^{2}≅\frac{K_{f}}{C_{ox}^{2}.WLf}$$ (9)

where Cox denotes the oxide capacitance, f represents the frequency, and K_f_ represents the flicker noise coefficient. It ranges from 10^−22^ to 10^−24^. Using Eq (10), the mean square input-referred noise voltage of the second-order source follower circuit is calculated

$$V_{in,n}^{2}=\frac{6.K_{f}{g_{m}}^{2}}{C_{ox}^{2}.f.W.L}\left\{\frac{r_{02}(g_{m}+{\left(r_{02}||r_{04}\right)}^{-1})}{g_{m}}\right\}$$ (10)

where the transistors M2 and M4’s respective output resistances are denoted by ro_2_ and ro_4_. The input and output referred to as noise are identical since the circuit’s gain is equal to unity. One can find the value of V^2^_in,rms_ for a given HD3 by using Eq (8). This is used in (10), and the result is an estimated dynamic range that is (11)

$$DR=10.{log}_{10}\left(\frac{V_{in,rms}^{2}}{V_{in,n}^{2}}\right)$$ (11)

### d. Minimum supply voltage required for the filter

Since the proposed design is targeted for low-power applications, the supply voltage should be as small as possible. The filter is implemented in 0.18*μ*m CMOS technology, Vt of 0.5V, maximum Vgs of 450mV, and voltage swing of 200mV. This implies that the transistors are in the weak inversion zone and increases the Vsat of PMOS transistors to 50 mV. Given these values, Eq (12) provides the minimal supply voltage needed for the PMOS source follower filter seen in Fig 2.

From Eq (12), it may be verified that Vdd, min should be at least 0.9V. A supply voltage of 1V is chosen for the design.


$$V_{dd,min}\geq {3v}_{sat}+v_{t}+v_{swing}$$ (12)

## III Tuning scheme based on magnitude comparison

**Fig 4:**
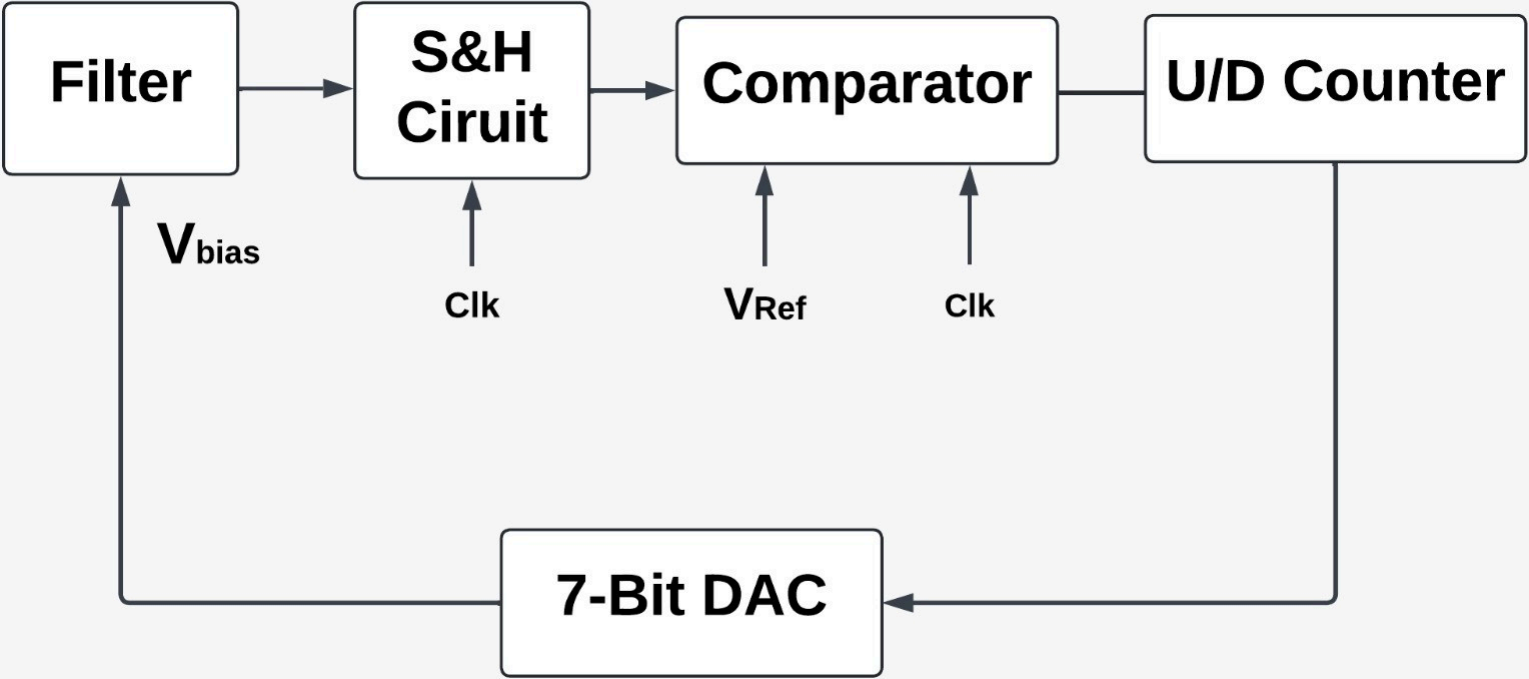
Block diagram of the tuning circuit.

The fabricated PMOS and NMOS transistors are categorized based on doping concentration changes in a silicon wafer (i.e) either as typical-typical (TT), slow-slow (SS), fast-fast (FF), slow-fast (SF) and fast-slow (FS). In this work, the integrating capacitor is a metal-in-metal (mimcaps) capacitor. It falls into one of the three categories mimcaps_typ (typical), mimcaps_max (maximum), and mimcaps_min (minimum). The supply voltage varies from the stated value. The circuit was designed at a temperature that may differ from the operational temperature. The fabrication-related uncertainties include aging, process, voltage, and temperature, which can change the time constant C/gm and, consequently, the cutoff frequency of the filter by 35%. A feature to adjust the filter parameters after fabrication should be included to achieve the desired cut-off frequency. One can accommodate for process variations and temperature dependencies by adjusting the transconductance value, capacitor value, or both. The tuning circuit proposed is shown in Fig 4. The components include a 7-bit Up-Down counter, a sample and hold circuit, a differential-matched comparator, a second-order source follower filter, and a &-bit current steering DAC. The 10pF capacitor is used in the sample & hold circuit.

### a. Principle of tuning

**Fig 5:**
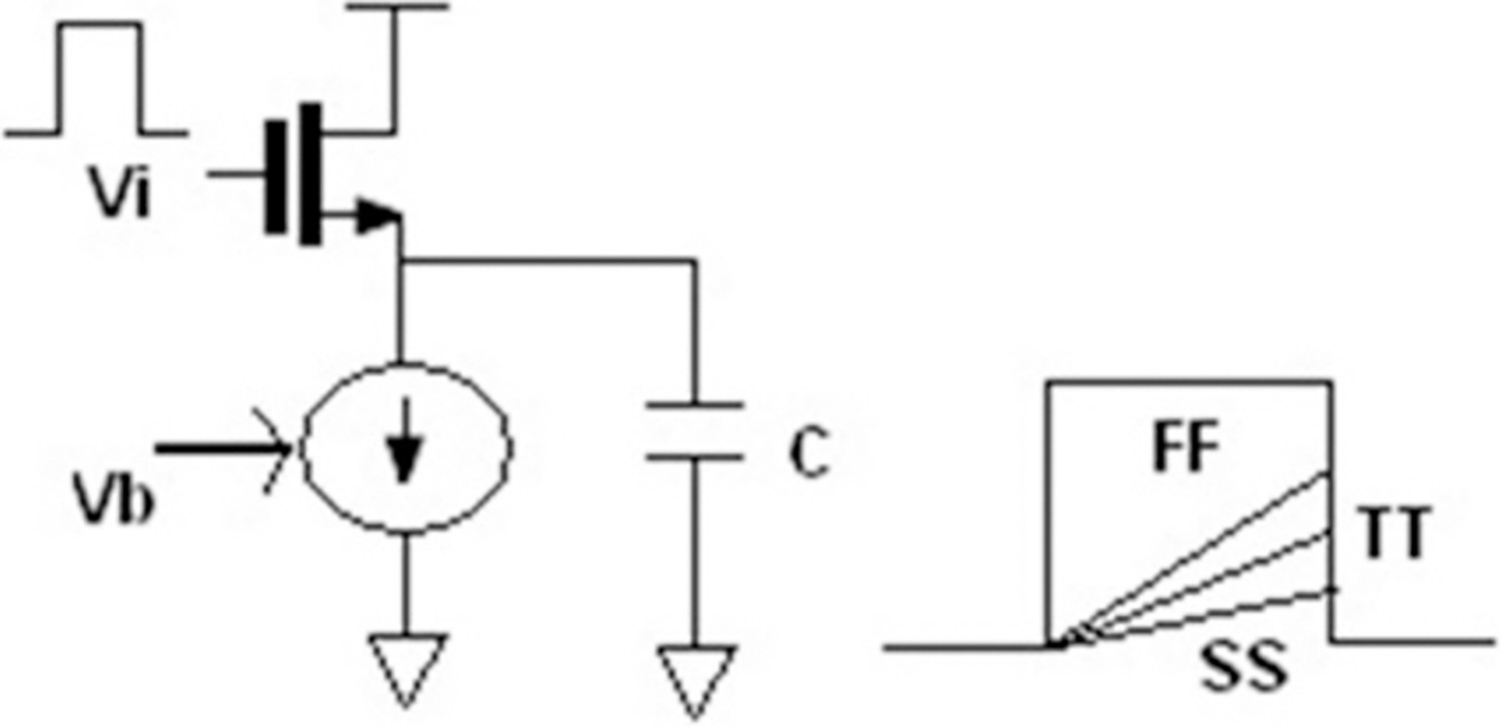
Output voltage of source follower across corners.

The source follower circuit is shown in Fig 5. Provide a discussion of the tuning concept. When the source follower receives a 1V step input, the capacitor can charge by the time constant C/gm. Assuming ideal conditions, the voltage across the capacitor at different time constants can be estimated. Let us assume the cutoff frequency of the filter to be 50Hz. For this cutoff frequency, the filter time constant is approximately 3.18ms. The voltage across the capacitor will increase up to 3-time constants. Therefore, during tuning, the duration “Ton” of the square input is chosen to be less than 3-time constants. A clock frequency of 100Hz will fulfill the requirement for this circuit. The voltage available across the capacitor at the falling edge of the clock input under the typical case is taken as the reference voltage (peak amplitude). Under normal operation, this reference voltage is compared with actual voltage across the capacitor at the falling edge of the clock and the bias voltage of the current source can be tuned till the reference voltage is attained. Using a grounded capacitor at the different ends is necessary for this scheme. This will remove the bottom plate capacitance effect but double the area of the capacitor.

### b. Circuit operation

**Fig 6:**
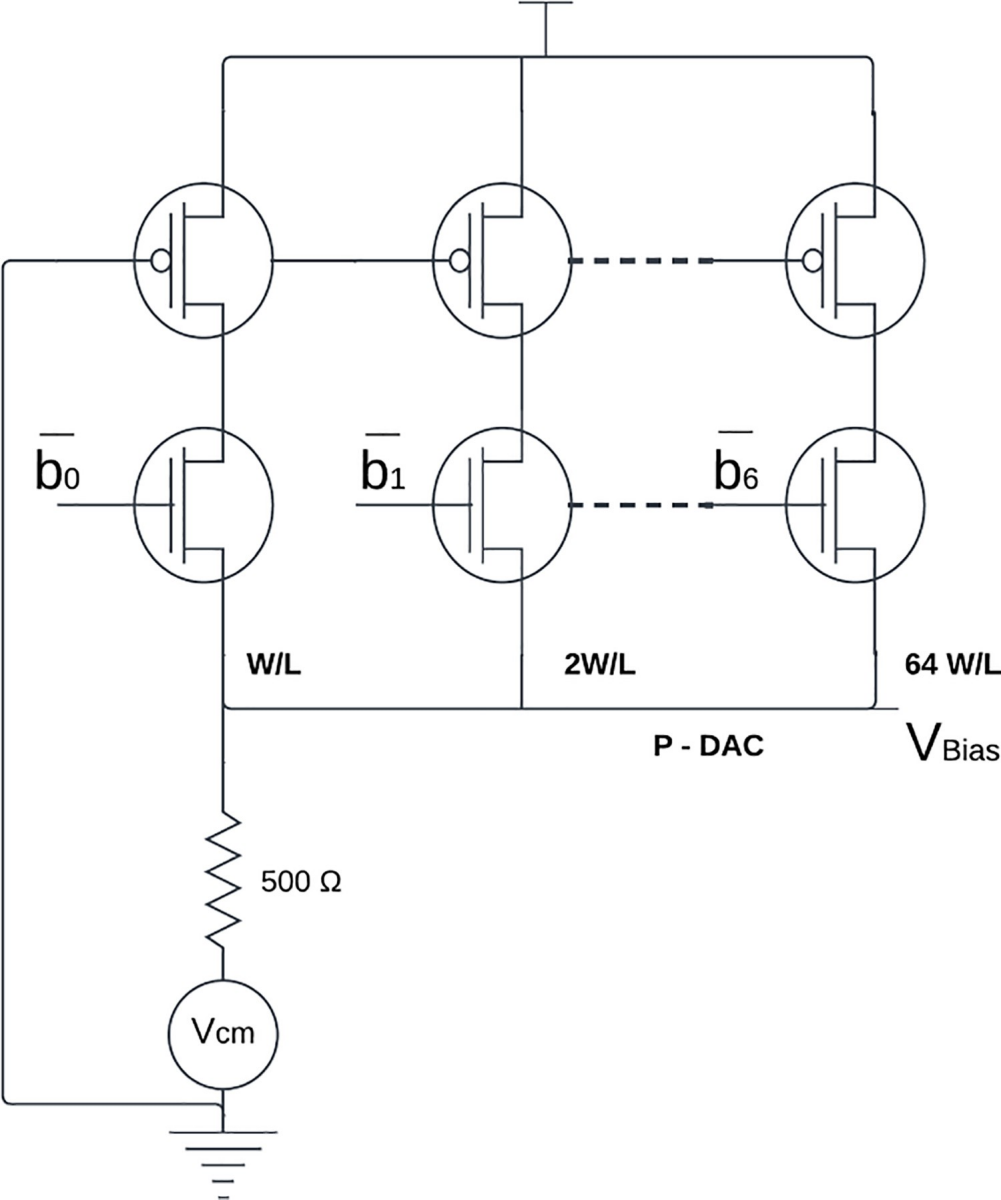
Circuit diagram of 7-bit current steering DAC.

**Fig 7:**
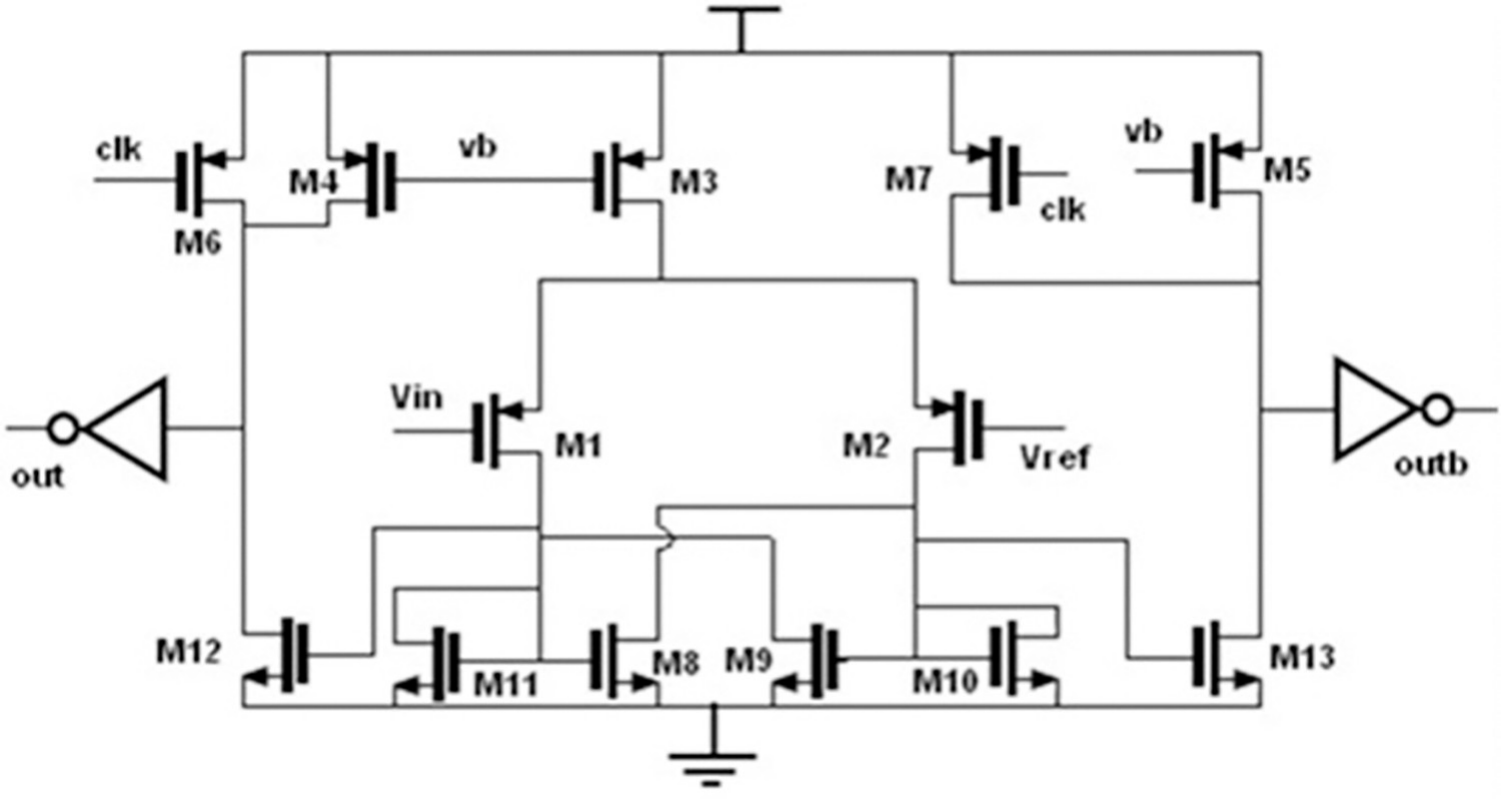
Circuit diagram of differential latched comparator.

By adjusting the bias voltages to the filter, the transconductance (g_m_) can be changed, which in turn can modify the cutoff frequency of the filter. As seen in Fig 6. a 7-bit CSDAC built with a PMOS transistor is used to generate the bias voltage. The digital inputs to the DACs are produced via the 7-bit UP/DOWN counter in Fig 4. The output of the differential latched comparator shown in Fig 7 determines the UP/DOWN mode. It is assumed for Fc = 50Hz, in this discussion. The capacitor (C) in the SS corner charges to a lower voltage (28 mV) than the reference voltage (80 mV). The down signal is produced by the comparator. At the same time, the DAC starts at the maximum value. the counter starts at zero. The filter’s time constant reaches its maximum at this voltage. The time constant of the filter drops as soon as the counter begins to reduce the count. The capacitor then charges in the direction of the reference voltage with each cycle. The comparator produces the up signal when the real voltage equals the reference voltage. Digital logic is used to stop the counter and latch the voltage at the DAC’s output based on changes in the comparator’s output.

In the FF corner. the comparator produces the up signal once the capacitor (C) charges to a higher value of 175mV. The minimal value is where the DAC starts, and the counter starts with all zeros. The voltage at which the filter operates has the lowest time constant. The filter structure’s time constant rises as the counter begins to increment the count. The capacitor charges towards the reference voltage. The comparator produces a down signal, the counter is halted, and the voltage at the DAC’s output is latching when the real voltage approaches the reference value. The real-time signals are applied to the filter once tuning is complete.

### c. DAC design

This work uses a 7-bit current steering digital to analog converter to generate the bias voltages. The bias voltage needed to produce a 50Hz cutoff frequency with standard corner devices is 650mV. The maximum Capacitance and SS transistors(mimcaps_max) raise the filter time constant. To achieve the typical cutoff frequency, the bias voltage must be lowered by 50 mV. The filter’s time constant also drops when FF transistors and minimum capacitance (mimcaps_max) are used. A 50mV bias voltage step from the nominal bias is required to reach the typical cutoff frequency. A DC voltage of 0.6V to 0.7V is therefore required. In Fig 6, the suggested DAC’s circuit diagram is displayed. Initially, the DAC circuit’s least important bit (b0) is selected. The current source transistor’s bias voltage is set to 0V. 3.6μm / 0.36μm is the selected W/L ratio for this transistor. For the switch to always operate in the saturation region, the W/L ratio of.24μm /.36μm was selected. The value of resistance that is chosen is 500 ohms. However, a DAC voltage range of 0.6V to 0.9V is needed to adjust the filter between 0.5Hz and 150Hz. The resistance value can be changed to achieve this. Similarly, the transistor sizes for the remaining DAC bits (b0-b6) are scaled appropriately. The DAC requires a 1V supply to function.

### d. Differential latched comparator

Fig 7 shows the differential comparator circuit diagram. The width of the input transistors is chosen to be large, such that the offset < 1mV. The output of the comparator is in the reset phase, (i.e.) two outputs out, out are low when the clock input is low. The comparison takes place during the logic 1 state of the clock. The dimensions of the transistors used in the comparator are shown in Table 1. The comparator operates on a 1V supply.

**Table 1:** Dimensions of latched comparator.

Transistors	Dimension of Transitors- W/L (all in μm)
M1,M2	25/0.7
M3,M4,M5,M6, M7	30/1
M8,M9	20/0.7
M10,M11	2/0.7
M12,M13	10/0.7

## IV Results and discussions

**Fig 8:**
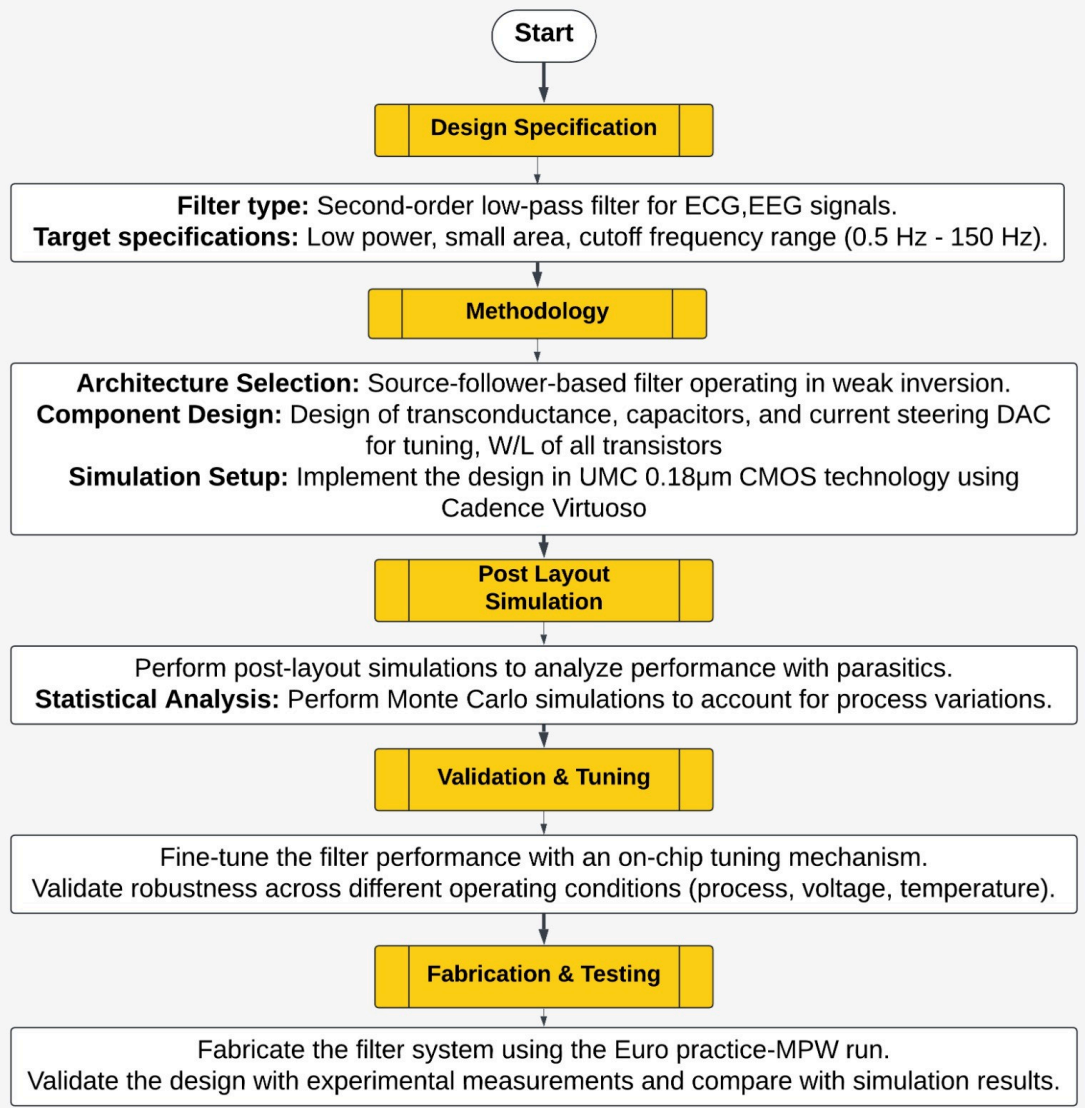
Flow diagram.

The tools and methods used for explained by flow diagram as shown in Fig 8.

**Fig 9:**
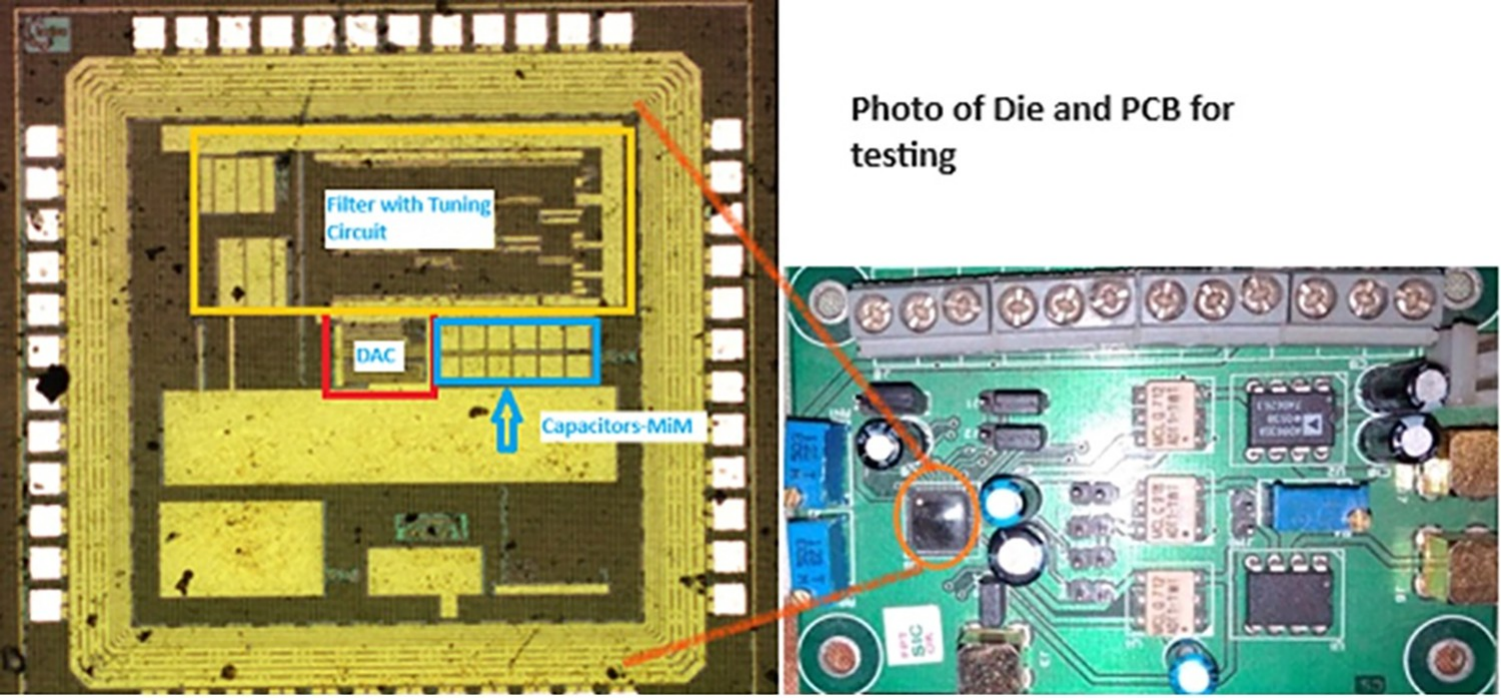
Microphotograph (left) of the proposed filter with tuning circuit and PCB test board (right).

The low power, programmable, 2^nd^ order low pass filter for biomedical signals is designed and implemented using UMC 0.18μm CMOS technology with 1 V supply. The filter was fabricated using MPW (Multi Project Wafer) run through Europractice tapeout services. The metal-in-metal capacitors (MIM) are used as their parasitic capacitances have a lesser effect on frequency response than those of double poly process. Cadence Virtuoso XL is used to draw the layout (IC6). Assura has been used to extract parasites, LVS, and DRC. For post-layout simulation, the Spectre simulator is employed. The microphotograph view of the filter with the tuning circuit and testing board for measurement of results is shown in Fig 9. The active area of the filter core including the tuning circuit occupies 0.065mm^2^. The filter is designed with a cut-off frequency programmable from 0.5Hz to 150Hz. From experimental results, the power dissipation of the filter core and the tuning circuit are found to be 6nW and 54μW respectively.

**Fig 10:**
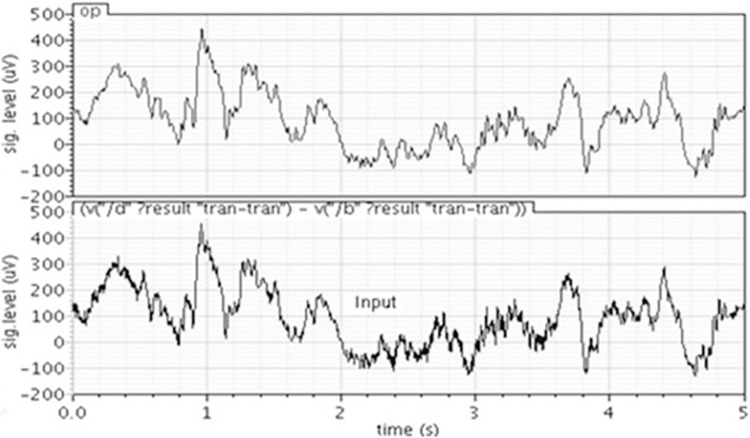
Output of the filter for EEG signal.

**Fig 11:**
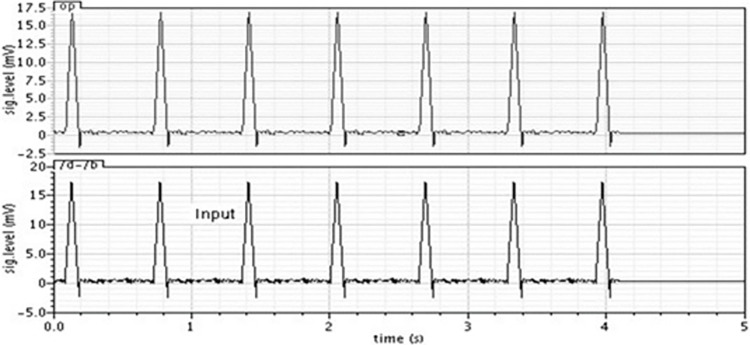
Output of the filter for ECG signal.

The ECG and EEG samples downloaded from [1,2] are high-pass filtered (to remove all frequencies from DC to 0.5Hz) using edf2wav converter. The time domain response of the filter for EEG and ECG signals, shown in Figs 10 and 11 respectively, demonstrates that the filter is working satisfactorily.

**Fig 12:**
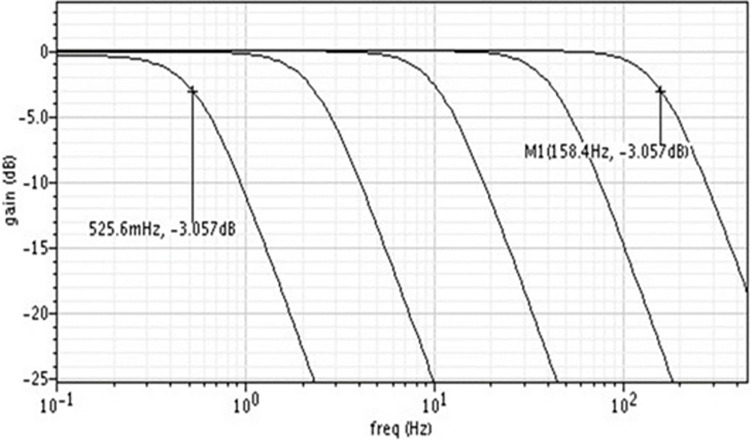
Magnitude response of the filter for various cutoff frequencies.

The magnitude response of the filter for various cutoff frequencies is shown in Fig 12.

**Fig 13:**
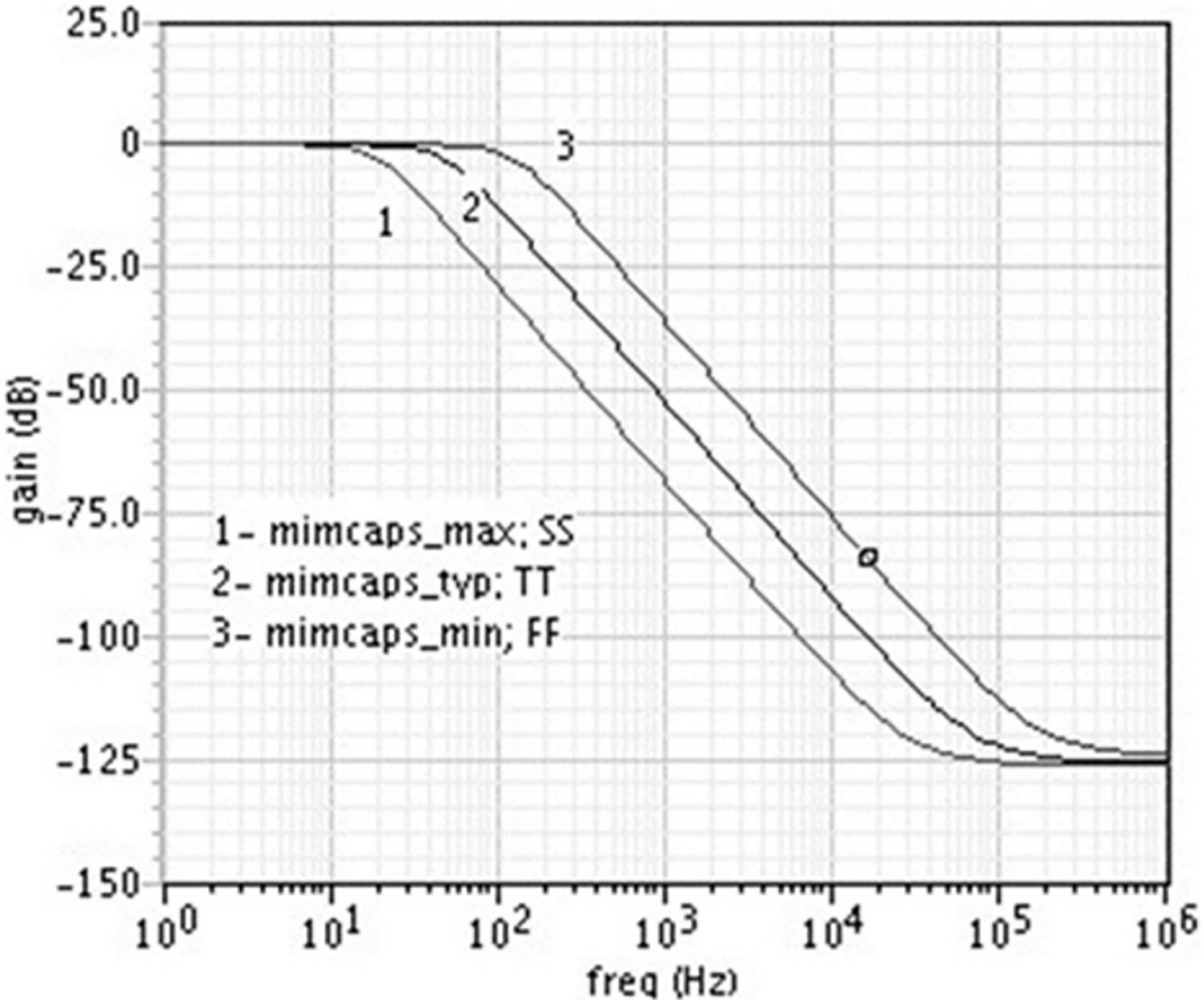
Magnitude response of the filter for various corners @Fc = 50Hz.

The magnitude response for various cutoff frequencies demonstrates the filter’s ability to adapt to different frequency requirements. The sharp roll-off beyond the cutoff frequencies indicates effective filtering of unwanted high-frequency components, ensuring that the signal within the desired frequency range is preserved accurately. This adaptability is crucial for biomedical applications where the signal frequency range can vary depending on the specific physiological measurement (EEG, EMG, ECG). We find that the DAC voltage range needed is 625mV to 800mV to set the cut-off frequency of the filter in the range of 0.5Hz to 150Hz. Fig 13 shows the filter’s magnitude response at Fc = 50Hz for various corners.

**Fig 14:**
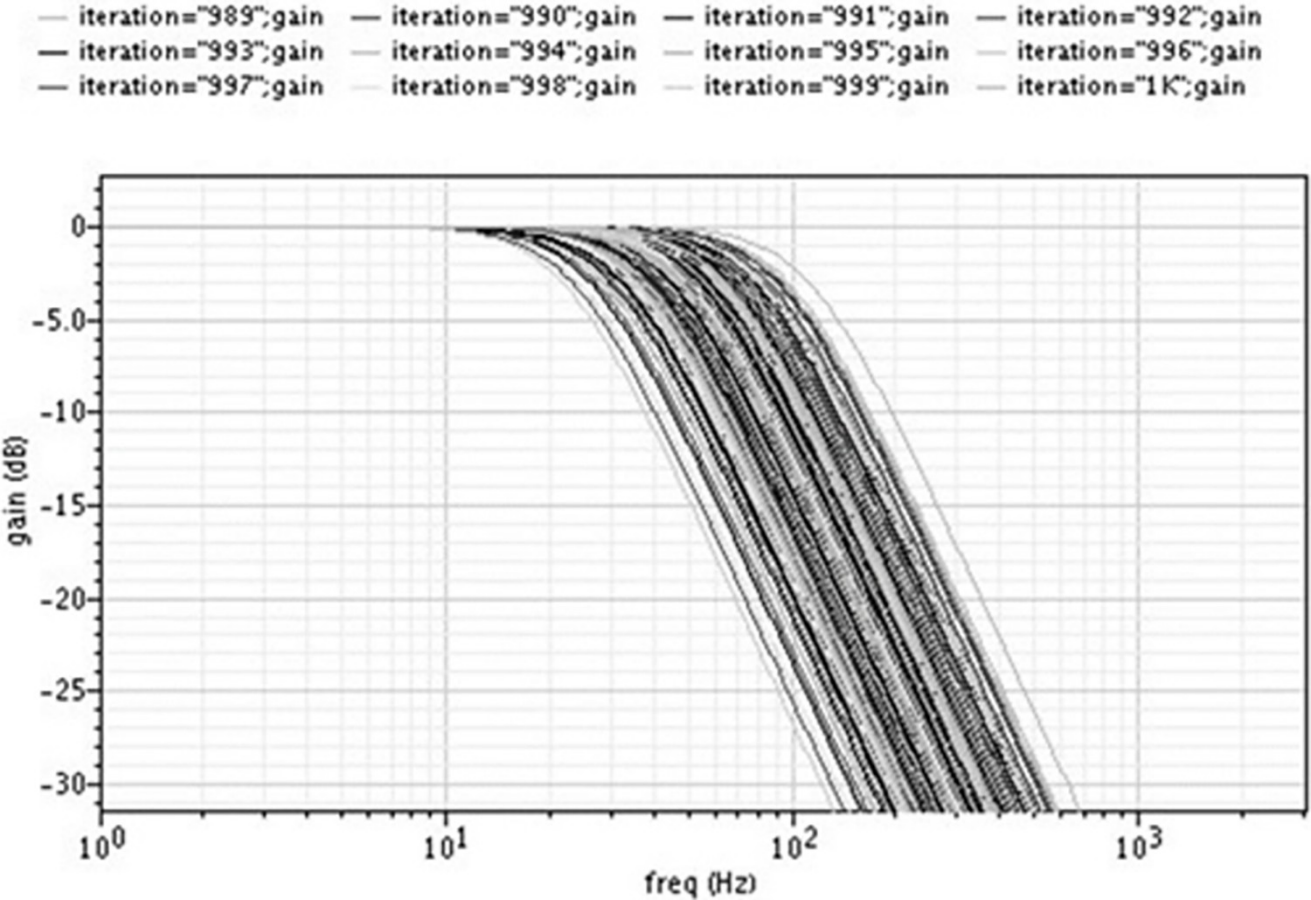
Montecarlo simulation (3 sigma).

Fig 14 displays the results of the Monte Carlo simulation for the filter with a bias voltage of 650mV (i.e., Fc = 50Hz) and with 3sigma variations for 1000 runs to understand the process variations.

The Monte Carlo simulation results illustrate the filter’s performance under process variations. The 3-sigma spread shows how much the center frequency is drifting due to process variations. This reliability under variation is critical for practical deployment.

From Figs 13 and 14, it may be noted that the center frequency of the filter varies from 22Hz to 120Hz due to process variations.

To understand the statistical variations better, the post layout simulations were performed on the designed filter with various process mismatch transistors for voltage and temperature variations. The designed filter was simulated under different voltage levels (e.g., ±10% of nominal voltage) and across a wide range of temperatures (e.g., from -40°C to 85°C). The obtained filter’s performance metrics such as cutoff frequency, power consumption, dynamic range, total harmonic distortion (THD), noise floor, and input referred noise are shown in Table 2.

**Table 2:** Performance metrics of filter for statistical variations.

Parameter	Simulated (TT) at 25°C, 1V	Simulated (TT) at -40°C, 0.9V	Simulated (TT) at 85°C, 1.1V	Simulated (SS) at 25°C, 1V	Simulated (FF) at 25°C, 1V
**Cutoff Frequency** (Hz)	50 Hz	43 Hz	57 Hz	46 Hz	54 Hz
**Power Consumption** (nW)	6 nW	5.5 nW	6.6 nW	5.8 nW	6.2 nW
**Dynamic Range** (dB)	75 dB	69 dB	71 dB	73 dB	72 dB
**THD** (dB)	-40 dB	-35 dB	-38 dB	-37 dB	-36 dB
**Noise Floor** (dBV/Hz)	-80 dBV/Hz	-75 dBV/Hz	-76 dBV/Hz	-78 dBV/Hz	-82 dBV/Hz
**Input-Referred Noise** (μVrms)	18 μVrms	16.4 μVrms	17 μVrms	18.6 μVrms	19.5 μVrms

**Fig 15:**
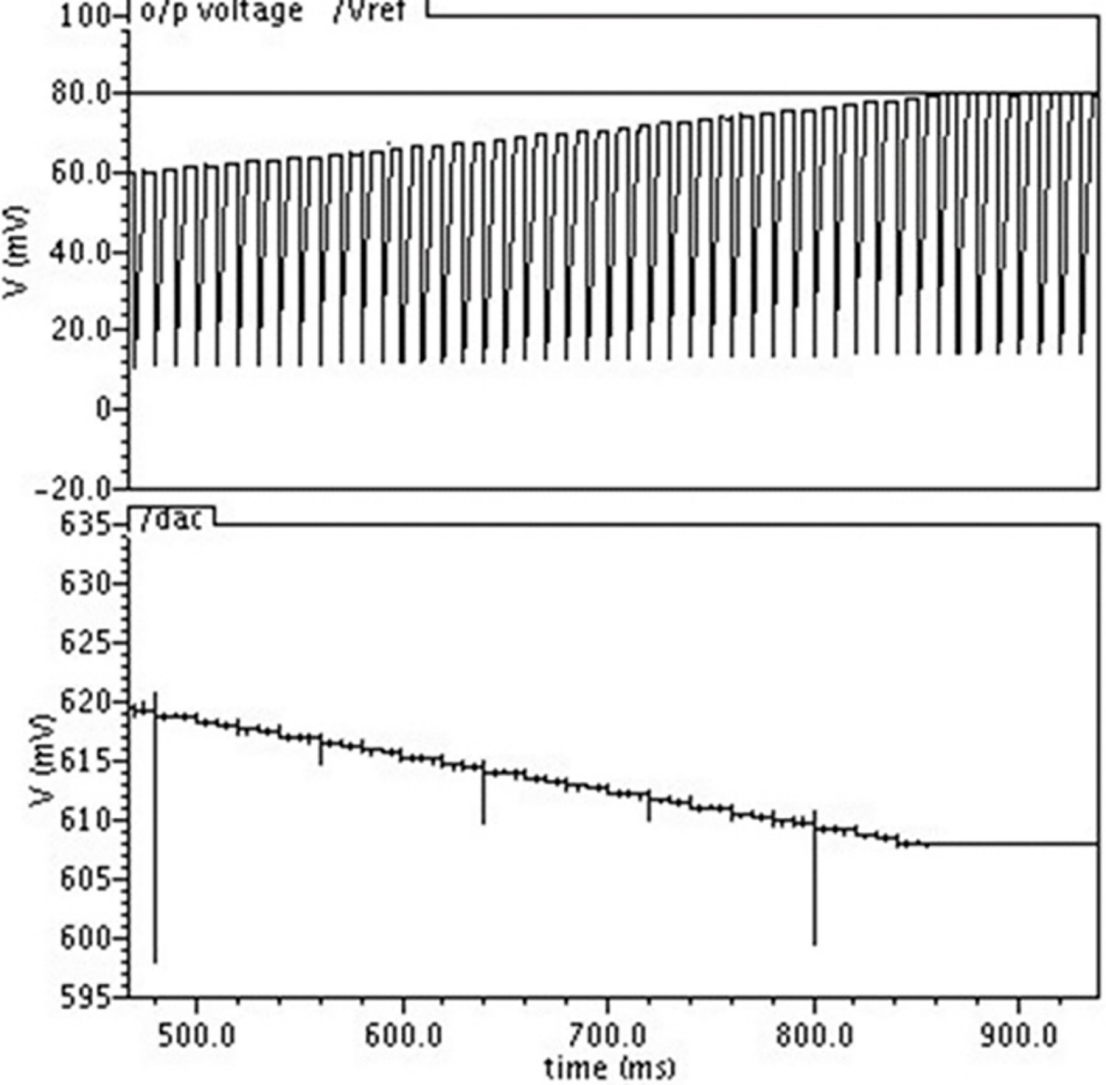
DAC and capacitor voltage at the end of the tuning process for worst-case condition1.

The insights from the above table indicate the robust PVT tuning mechanism is needed in order to maintain the spread. To obtain the desired cutoff frequency of say 50Hz, the filter has to be tuned. A cutoff frequency of 22Hz corresponds to the worst-case condition1 (wc1-SS transistor, mimcaps_max) and 120Hz corresponds to other worst-case condition2 (wc2-FF transistor, mimcaps_min). The tuning circuit is tested by simulating the circuit with worst-case condition wc1. Fig 15 shows the simulation results.

**Fig 16:**
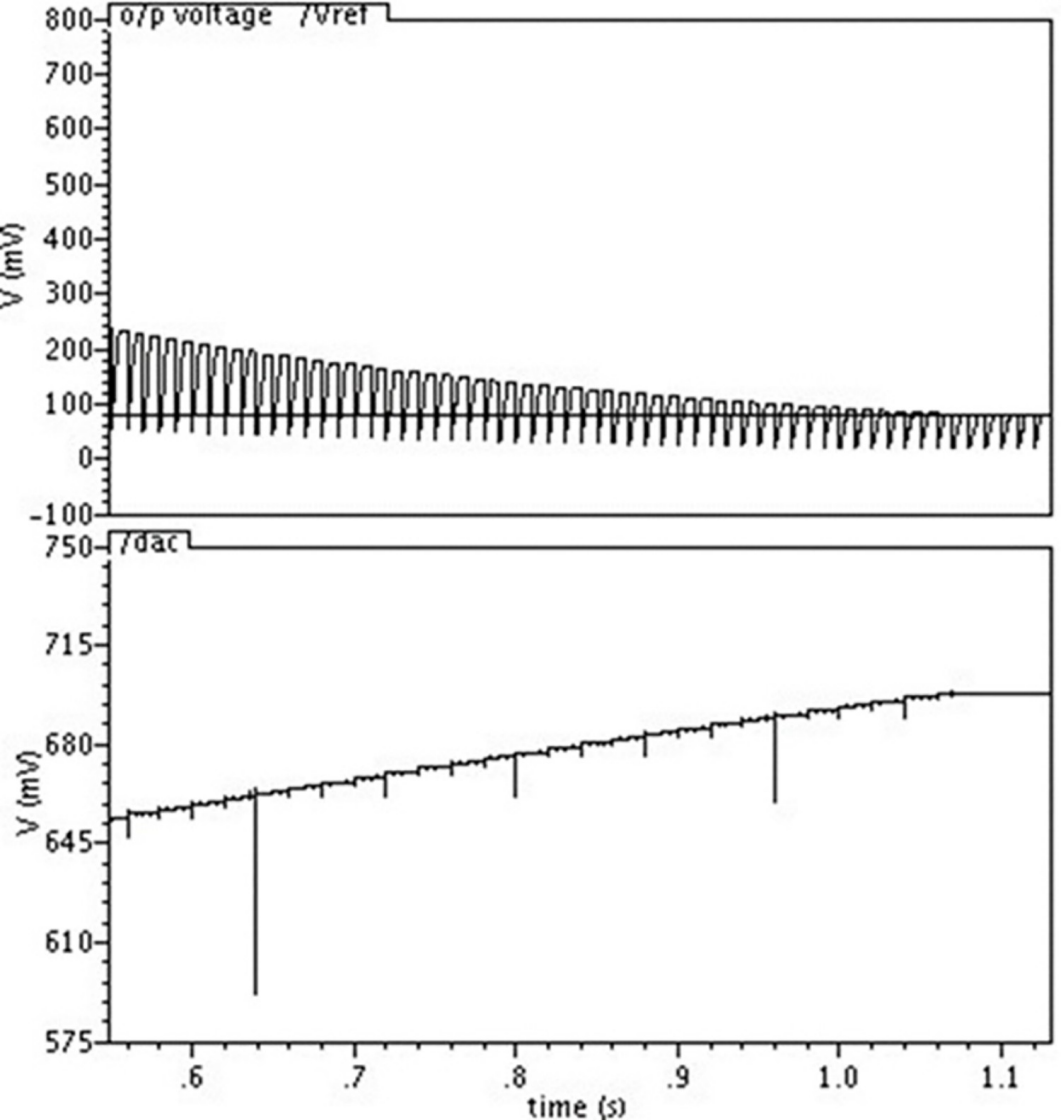
DAC and capacitor voltage at the end of the tuning process for worst-case condition2.

It is evident that at the beginning of the simulation, the voltage at the capacitor is 60. It suggests that the circuit’s time constant is high. 80mV is the reference voltage, as the tuning goes on, the AC voltage drops (giving the current source more bias), and it stops as soon as the capacitor voltage reaches 80mV. A DAC voltage of 608mV is adequate to achieve a 50Hz cutoff frequency. Similarly, another worst-case scenario (wc2) is simulated to verify the tuning circuit. Simulation results are displayed in Fig 16.

**Fig 17:**
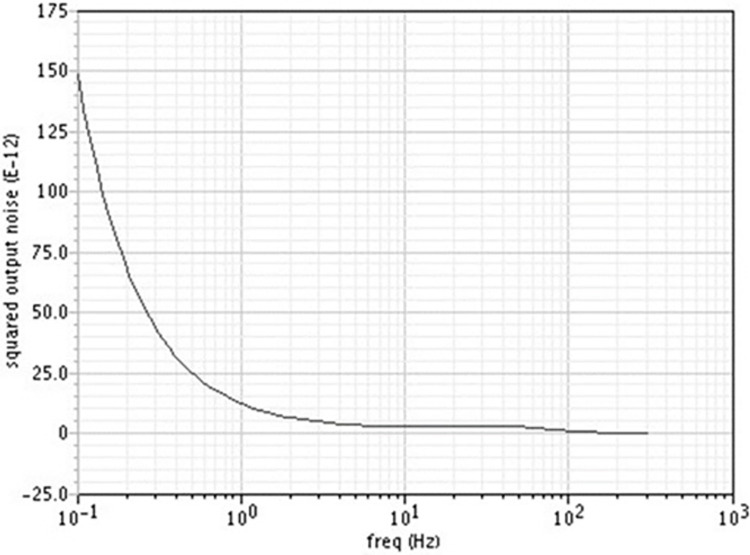
Simulated noise power spectrum for 15Hz sine wave (a) Vin = 40mV (b) Vin = 60mV (c) Vin = 80mV.

As can be seen, at the beginning of the simulation, the voltage at the capacitor is 240 mV. As tuning proceeds, the DAC voltage rises(supplies less bias to the current source) and stops as soon as the capacitor voltage reaches 80mV. To achieve the 50Hz cutoff frequency, a DAC voltage of 690mV is adequate for the straightforward reason that the transconductance value drops with temperature and mimics the properties of SS transistors and vice versa, the tuning circuit is not tested at different temperatures in this instance. The DAC resolution and comparator accuracy control the automated on-chip tuning’s accuracy. The DAC’s LSB resolution in this case is 0.8 mV. The precision of the filter tuning is 2%. The tuning process analysis under worst-case conditions demonstrates the filter’s capability to adjust to different scenarios. The DAC and capacitor voltage levels indicate successful tuning, ensuring optimal filter performance. This adaptability is vital for maintaining signal integrity across a wide range of conditions, thereby enhancing the robustness of the biomedical signal filtering system.The noise spectrums of the filter for the sinusoidal signal of 15Hz with various amplitudes are shown in Fig 17. It can be observed from simulations that the noise floor is around -80dB.

The noise power spectrum for a 15Hz sine wave at various input voltages showcases the filter’s noise-handling capabilities. The results indicate that the filter effectively suppresses noise, maintaining a clear signal across different input levels. This noise suppression is crucial for accurate biomedical signal interpretation, where noise can obscure critical physiological information.

**Fig 18:**
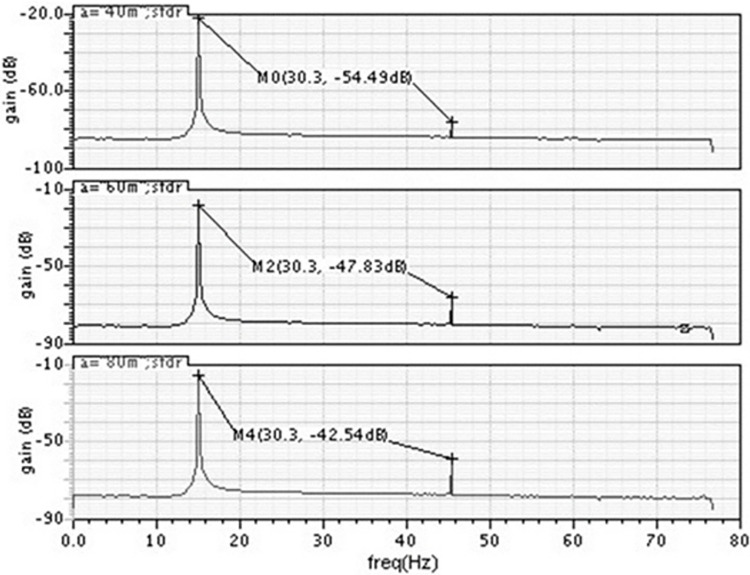
Output noise voltage density.

The output noise voltage density of the filter up to 300Hz is shown in Fig 18. Being the source follower, the gain of the filter is 1. The integrated input referred to noise up to 300Hz is 18μVrms.

The output noise voltage density provides a quantitative measure of the noise performance of the filter. The low noise density values affirm the filter’s ability to minimize noise interference, thus preserving the quality of the biomedical signals. Low noise is particularly important in biomedical applications where signal precision can directly impact diagnostic outcomes.

**Fig 19:**
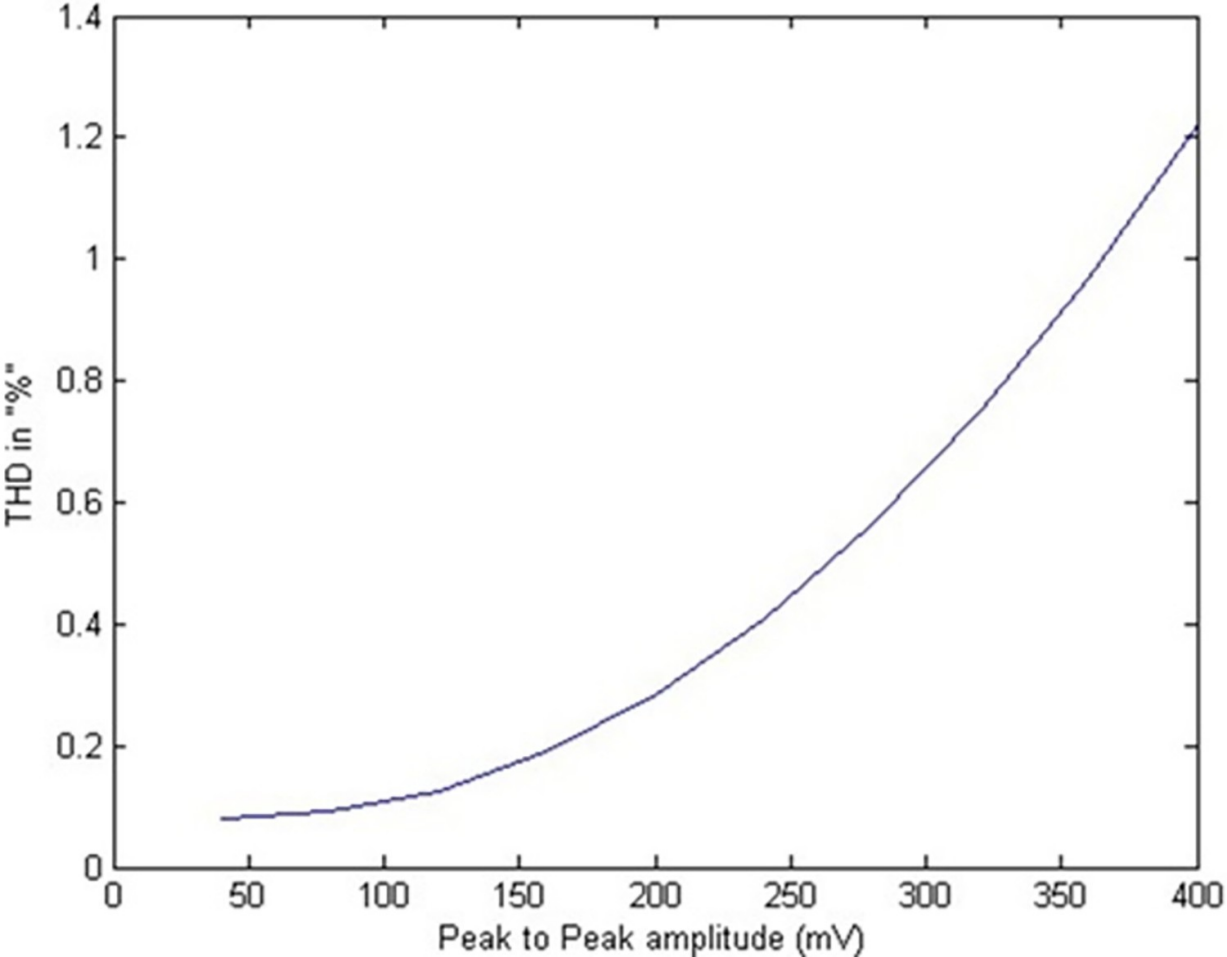
THD Vs input voltage.

**Fig 20:**
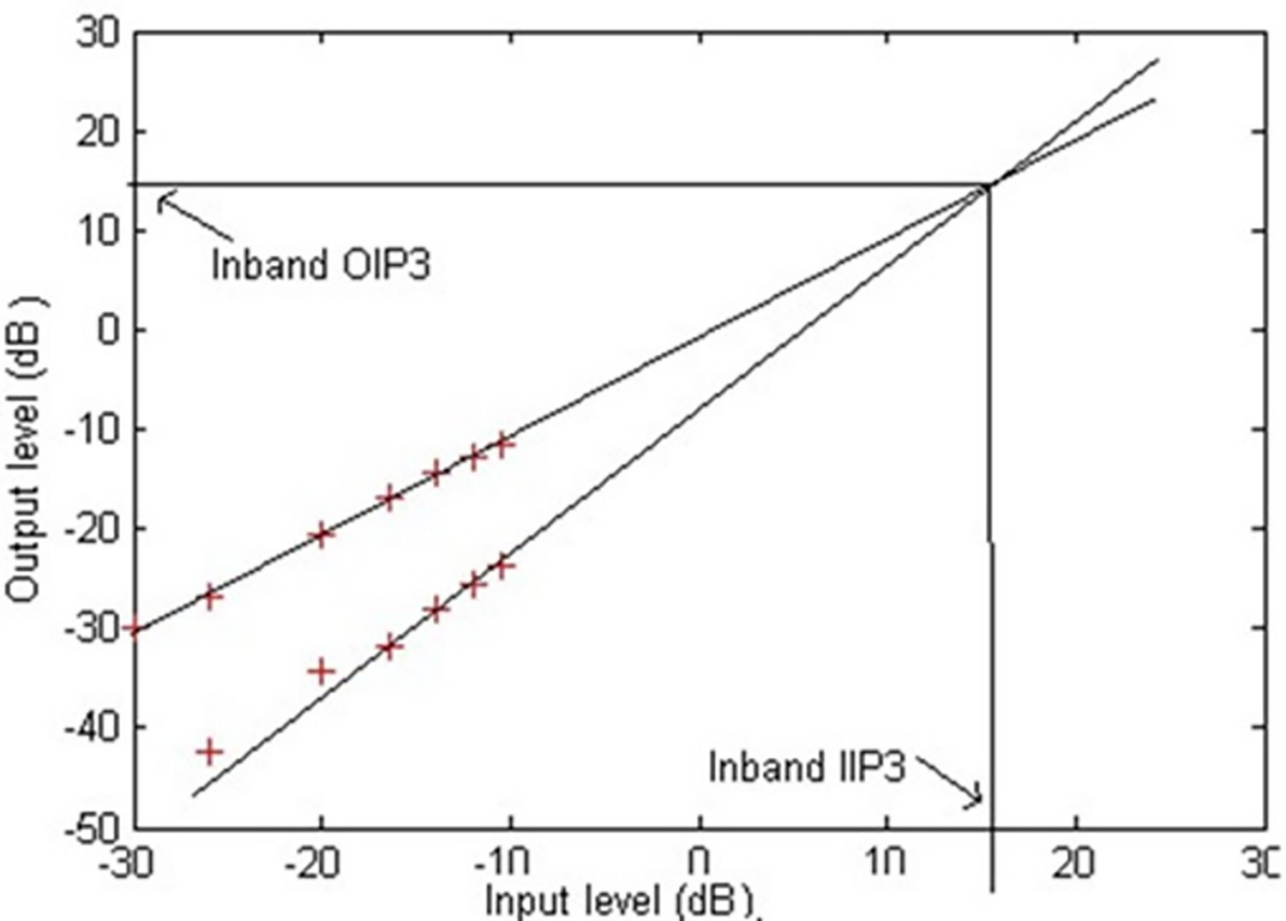
IIP3 plot with inband tones at 13Hz and 15Hz.

The total harmonic distortion (THD) versus peak-to-peak differential input voltage plot is shown in Fig 19. It can be noted from this Fig that the upper bound on THD is -40dB (1%) for 380mVpp signal swing. From the ratio of the rms voltage of noise and signal, the dynamic range is computed to be around 75 dB. The Total Harmonic Distortion (THD) versus input voltage plot indicates the filter’s linearity and distortion characteristics. Low THD values across a range of input voltages suggest that the filter introduces minimal harmonic distortion, which is crucial for maintaining the integrity of the biomedical signals. Fig 20 shows an IIP3 (Third order Input Intercept Point) simulation using two in-band tones that are close to each other (13Hz and 15Hz). An IIP3 of 16 dBVp and OIP3 of 15 dBVp is achieved.

**Fig 21:**
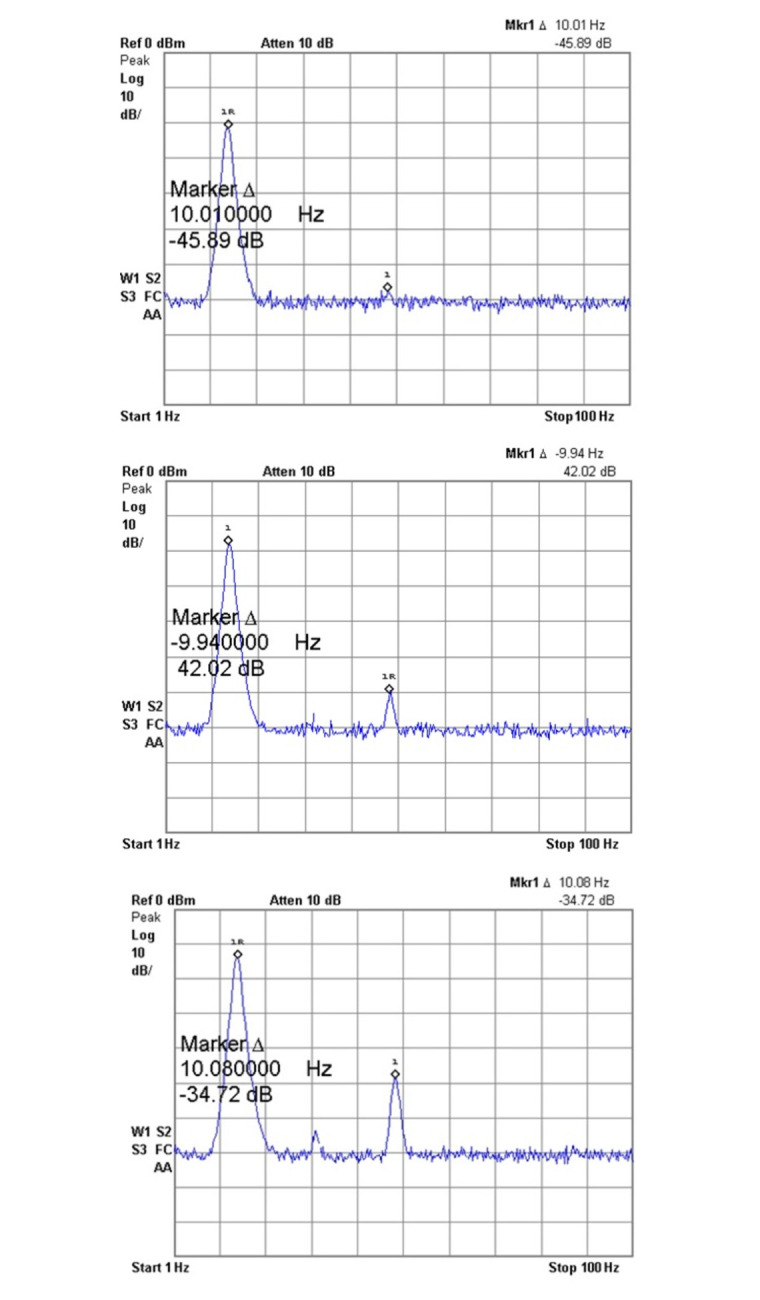
IIP3 plot for: (a) 80mVpp (b) 120mVpp (c) 300mVpp.

The third-order intercept point (IIP3) plot demonstrates the filter’s intermodulation performance. High IIP3 values indicate excellent linearity and low intermodulation distortion, ensuring that the filter can handle inband tones effectively without introducing significant distortion. This capability is important for complex biomedical signals where multiple frequency components must be accurately processed. Tektronix spectrum analyzer (RSA 5103 B) was used to measure the spurious-free dynamic range (SFDR) of a second-order source follower filter at three different input signal amplitudes: 80mVpp, 120mVpp, and 200mVpp. The SFDR plots of the same are shown in Fig 21. Since the fabricated filters are used for EEG and ECG signal processing, the measurements were carried around 10Hz. SFDR is the ratio between the power of the fundamental signal and the power of the strongest spurious signal within the bandwidth of interest. For the 80mVpp input signal, the SFDR can be calculated from the difference between the fundamental tone at 10.01 Hz and the next largest spurious tone, and from Fig 21(A), it is observed to be 45.89 dB. Similarly, for the 120mVpp input signal, the SFDR can be calculated from Fig 21(B), it is observed to be 42.02 dB. In the same way for the 300mVpp input signal, the SFDR can be calculated from Fig 21(C), it is observed to be 34.72 dB. The second-order source follower filter exhibits different performance characteristics based on the input signal amplitude. At lower amplitudes, the filter maintains a cleaner output with a higher SFDR. However, as the input amplitude increases, the SFDR decreases due to increased harmonic distortion and non-linearities in the system. This analysis is crucial for applications where maintaining high signal fidelity is essential, and it highlights the importance of operating the filter within its optimal amplitude range to ensure minimal spurious signals.

**Fig 22:**
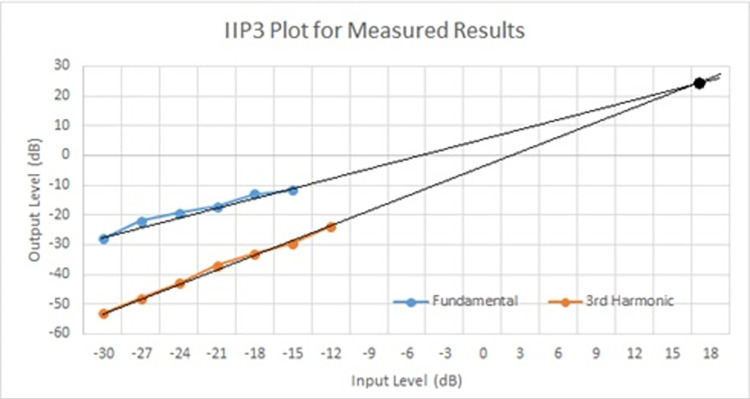
Measured results of IIP3 plot.

Fig 22 shows an IIP3 (Third order Input Intercept Point) using the measured results of the second-order source follower filter. This graph provides a way to evaluate the linearity and distortion performance of the filter circuit. The fundamental signal (Blue line) represents the output level of the fundamental frequency as the input level increases. It generally shows a linear relationship, indicating that the output increases proportionally with the input. The fundamental signal (Blue line) represents the output level of the fundamental frequency as the input level increases. It generally shows a linear relationship, indicating that the output increases proportionally with the input. The third harmonic (Orange line) represents the output level of the third harmonic distortion. The slope of this line is usually 3:1, meaning the third harmonic grows three times faster than the fundamental frequency as the input increases. The third harmonic is a critical measure of nonlinearity and intermodulation distortion in the system. The IIP3 is found at the point where the extrapolated linear trends of the fundamental and the third harmonic intersect. In this Fig, the intersection occurs around an input level of approximately 16 dB. This is the IIP3 value, indicating that at this input power level, the power of the fundamental and the third-order intermodulation product would be equal.

The performance summary of the proposed filter is listed in Table 3.

**Table 3:** Performance summary.

Parameters	Value
Technology	CMOS 0.18μm
Filter	2nd order Butterworth
Supply voltage	1V
IIP3	16dBVp
Input referred noise (up to 300Hz)	18μVrms
BW (Programmable)	0.5 Hz– 150Hz
Active area	0.065mm^2^
Power	6nW–Filter core 54μW–Tuning circuit
Vinpp @ THD = -40dB	300mVpp
DR@ THD≤-40dB	75dB

The performance comparison between this work and the previous lowpass Gm-C filters for low-frequency ranges is given in Table 4. In this discussion, we will focus on the performance metrics: Figure of Merit (FoM), Normalized Area (NA), and Normalized Power (NP) of the proposed biomedical filter compared to other filters reported in the literature. Two FoMs are introduced, FoM1, and FoM2 [3], which use normalized power and other calculated for normalized area. The unit for Figure of Merit is Joules(J). All the metrics have to be the lowest for the filter architecture. It is important to note that, the proposed filter includes a tuning circuitry designed to compensate for process variations, ensuring consistent performance. The work from [13] (2015) has the smallest area at **0.04 mm^2^**, which makes it highly area efficient. However, this comes with lower power consumption and dynamic range. Among the listed works, only reference [15] and this work include chip measurement results. The key performance metrics such as bandwidth (BW), dynamic range (DR), power consumption, and figure of merit (FoM) are discussed below to highlight the superiority of this work.

**Table 4:** Performance comparison of the proposed filter with other filters.

Parameter	2015	2019	2020	2020	2022	2023	This^@^
	[13]	[14]	[15]- M/E	[17]	[18]	[16]	Work-M/E
VDD	1.5V	1.8V	1.8V	0.5V	0.5V	0.5V	1V
Tech	CMOS	CMOS	CMOS	CMOS	DTMOS	CMOS	CMOS
	0.35μm	0.18μm	0.18μm	0.13μm	0.18μm	0.18μm	0.18μm
Type	Gm-C	Gm-C	OTA-C	Gm-C	OTA-C	Gm-C	Gm-C
	(W.I)	(W.I)		(W.I)		(W.I)	(W.I)
Vth	0.6V	0.6V	0.6V	0.35V	0.4V	0.35V	0.5V
Order	2(S)	2(D)	2(D)	4(D)	5(D)	4(D)	2(D)
BW	250Hz	100Hz	300Hz	50Hz	50Hz	100Hz	150Hz
DR	59.6dB	91.86dB	76dB	65.17dB	49.7dB	57.14dB	75dB
Power	1.9nW	25.95nW	25 μW	2.46nW	17.5 nW	0.4nW	6nW
Area	0.04mm^2^	0.121 mm^2^	0.068 mm^2^	0.0996 mm^2^	NA	0.052 mm^2^	0.065 mm^2^
NP*	0.703x10^-9^	6.006x10^-9^	5.787x10^-6^	16.4x10^-9^	175 x 10^−9^	2.66x10^-9^	6x10^-9^
NA^+^	0.326 x10^6^	3.73x10^6^	2.09x10^6^	5.893x10^6^	--	1.604x10^6^	2.00x10^6^
FoM1^%^	2.35x10^-14^	3.26x10^-13^	1.27x10^-10^	1.26x10^-13^	14.08x10^-13^	1.16x10^-13^	2.66x10^-13^
FoM2^^^	1.09x10^-1^	2.03x10^-2^	4.58x10^-1^	4.52x10^-1^	--	7.01x10^-1^	8.88x10^-1^

%—FoM1 = NP/ (Order x BW x DR).

^—FoM2 = NA / (Order x BW x DR).

*—Normalized Power: NP = Power x (0.5/(Vdd—Vth)) x 1/Vdd.

+—Normalized Area: NA = Area/Tech2.

@- Includes Compensation Circuit for PVT tuning.

### Power consumption

The proposed filter achieves an impressive power consumption of 6nW, which is substantially lower than the 25 μW reported in [15]. This significant reduction in power consumption makes this filter highly suitable for portable biomedical applications, where battery life is critical. Compared to [15], which consumes nearly 4,166 times more power, the proposed work demonstrates a superior design for ultra-low-power operation.

### Bandwidth and dynamic range

The filter offers a bandwidth of 150 Hz, covering a broader range than the 100 Hz of [15]. This broader bandwidth makes it more versatile for biomedical applications that require processing signals with varying frequencies, such as ECG and EEG. Furthermore, the filter achieves a dynamic range (DR) of 75 dB, which is competitive and on par with the 76-dB reported in [15]. This balance between bandwidth and dynamic range ensures that the filter can process a wide range of signals while maintaining high signal fidelity.

### Area efficiency

In terms of silicon area, the proposed filter occupies 0.065 mm^2^, which is slightly smaller than the 0.068 mm^2^ of [15]. Although the difference in area is marginal, this slight reduction in area, combined with significantly lower power consumption, highlights the efficiency of the design.

### Figure of merit (FoM)

The normalized power figure of merit (FoM1) for the proposed filter is 2.66 × 10⁻^13^ J, which is notably better than the 1.27 × 10⁻^1^⁰ J reported in [15]. This substantial improvement in FoM1 reflects the optimized power efficiency of the filter. The FoM2, which considers both area and performance, is 8.88 × 10⁻^1^ for the proposed filter. While [15] achieves a better FoM2 of 4.58 × 10⁻^1^, it comes at a significantly higher power cost, making the proposed design more favorable in low-power scenarios.

The proposed work outperforms the chip results from [15] in terms of power efficiency, achieving a remarkable balance between bandwidth, dynamic range, and silicon area. This combination of low power consumption and compact area makes the filter an ideal candidate for portable biomedical devices, particularly in applications where prolonged battery life is essential. Overall, the superiority of this work lies in its ability to deliver high performance while consuming minimal power, making it a standout solution in the field of low-power biomedical filters.

### Limitations of this work

Operating in the weak inversion region presents design challenges, including maintaining linearity and managing noise. The trade-offs between power efficiency and other performance metrics, such as THD and dynamic range, need careful balancing, which can complicate the design process.

The input-referred noise and the noise floor can still be impacted by temperature and process variations. This could limit the filter’s effectiveness in environments with high interference or when processing extremely weak biomedical.

The current design is focused on a second-order filter. For more complex signal processing tasks, higher-order filters may be required, which would increase the design complexity.

## V Conclusions

In this work, a low-power, programmable second-order Gm-C low-pass filter was successfully implemented using standard 0.18μm CMOS technology, targeting biomedical applications such as ECG and EEG signal processing. The filter demonstrates excellent performance, with a tunable cutoff frequency range of 0.5 Hz to 150 Hz, minimal power dissipation of 6nW, and a compact silicon area of 0.065 mm^2^. The achieved dynamic range of 75 dB, along with the integrated on-chip tuning mechanism, ensures stable operation across process, voltage, and temperature variations. These features make the proposed filter highly suitable for portable biomedical devices where power efficiency and robust performance are critical. Practical implications of this work include its applicability in low-power wearable and portable biomedical systems, which require continuous operation without frequent battery replacements. Current limitations include the need for further optimization to enhance performance in extreme process variations and temperature conditions. Future work will focus on extending the design to higher-order filter structures and exploring more advanced CMOS technologies to further reduce power consumption and improve performance in ultra-low-power biomedical applications.

## Supporting information

S1 File File containing the schematic, layout of biomedical filter with tuning circuit using Cadence toolsack. (ZIP)
